# Exploring Single-Molecular Magnets for Quantum Technologies

**DOI:** 10.3390/molecules30122522

**Published:** 2025-06-09

**Authors:** Wei Wu, Tianhong Huang, Jianhua Zhu, Taoyu Zou, Hai Wang

**Affiliations:** 1 UCL Department of Physics and Astronomy, University College London, Gower Street, London WC1E 6BT, UK; 2 Yunnan Key Laboratory of Metal-Organic Molecular Materials and Devices, Kunming University, Kunming 650214, China; 3 Key Laboratory of Yunnan Provincial Higher Education Institutions for Organic Optoelectronic Materials and Devices, Kunming University, Kunming 650214, China; 4 School of Physical Science and Technology, Kunming University, Kunming 650214, China; 5 School of Physics, Peking University, Chengfu Road 209, Beijing 100871, China; 6 Department of Chemical Engineering, Pohang University of Science and Technology, 77 Cheongam-Ro, Nam-Gu, Pohang 37673, Republic of Korea; 7 School of Physics and Astronomy, Center for Optoelectronics Engineering Research, Yunnan University, Kunming 650091, China

**Keywords:** single-molecule magnet, click chemistry, supramolecular chemistry, SQUID, TR-EPR, XMCD, quantum technologies, density functional theory, post-Hartree–Fock, theory of open quantum systems

## Abstract

A single-molecule magnet (SMM) is a molecule that functions as a magnet. SMMs can be explored not only for emerging technology but also the fundamental science of their quantum nature, nanometer sizes, and their ease of engineering. This review encompasses the state-of-the-art experiments and theories developed so far for SMMs. We briefly explore their experimental synthesis and characterization. In the experimental synthesis, we cover ‘Click Chemistry’ and supramolecular chemistry. The main experimental characterizations comprise superconducting quantum interference devices, electron paramagnetic resonance, neutron scattering, and X-ray magnetic circular dichroism. The theoretical and computational works based on the density functional theory, the post-Hartree–Fock methods, and the theory of open quantum systems are discussed. Moreover, we exemplify the numerous promising research areas for SMMs by discussing quantum technologies. We envision a brilliant future for the fundamental research and emerging applications of SMMs.

## 1. Introduction

**Figure 1:**
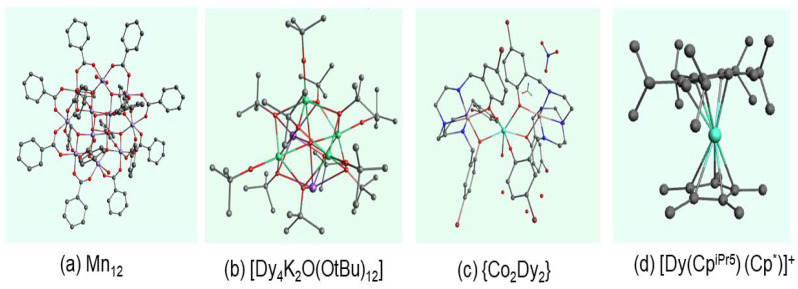
Typical examples of SMMs. (**a**) ${\mathrm{Mn}}_{12}$ [1], (**b**) polynuclear lanthanide SMM ${\mathrm{Dy}}_{4}$ [3], (**c**) 3d−4f SMM [4], and (**d**) lanthanide SIM [5]. Here, carbon is in gray, nitrogen in blue, oxygen in red, manganese in purple, bromine in dark red, dysprosium in green, and cobalt in dark pink.

As the name suggests, a single-molecule magnet (SMM) is a molecule that acts like a tiny magnet. SMMs have been developed since the first reported synthesis of [${\mathrm{Mn}}_{12}O_{12}$($O_{2}$CR)_16_($H_{2}$O)_4_] (${\mathrm{Mn}}_{12}$). ${\mathrm{Mn}}_{12}$ is a high-spin molecule displaying slow magnetic relaxation at low temperatures [1]. This type of molecule-based magnet was first named a *single-molecule magnet* after the fabrication of [${\mathrm{Mn}}^{\mathrm{IV}}{\mathrm{Mn}}_{3}^{\mathrm{III}}O_{3}$X] [2]. In Figure 1, we have shown some typical examples of ${\mathrm{SMMs—Mn}}_{12}$ (the SMM synthesized in the early 1990s) [1], ${\mathrm{Dy}}_{4}$ polynuclear lanthanide SMM [3], $3d-4f{\mathrm{Co}}_{2}{\mathrm{-Dy}}_{2}$ SMM [4], and the Dy(III) single-ion magnet (SIM) that contains only one magnetic metal ion [5].

**Figure 2:**
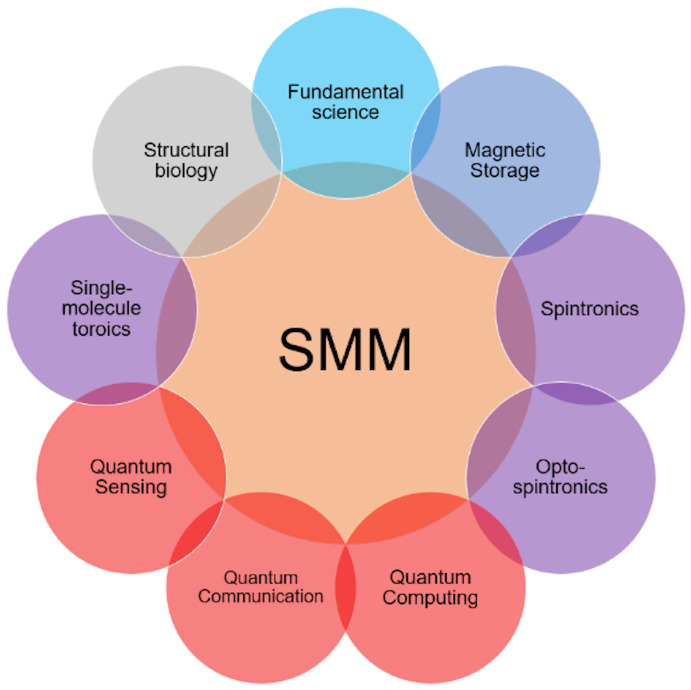
A summary of important scientific and application potentials for SMMs.

SMMs have attracted unprecedented research interest since their birth, owing to their promise for many research areas, such as magnetic storage, spintronics, quantum technologies, and structural biology [1,2,6,7,8,9,10,11,12,13,14,15,16] (Figure 2). Moreover, SMMs could play an important role as a platform for studying fundamental quantum science through a large variety of spin models and the flexibility of ligand engineering. The material demands of emerging technologies [10,12] provide fantastic opportunities to physicists and chemists to model, design, and synthesize new SMM species.

SMMs are also suitable for the study of quantum tunneling between different magnetic states (bi-stable states), raising intensive interest in molecular quantum science [17,18]. SMMs can be used as the molecular quantum bit (qubit) that has a two-dimensional Hilbert space. If the SMM has a stable spin state higher than $\frac{1}{2}$, it can even work as a qudit with *d* dimensions (normally d>2). This could offer a series of benefits for quantum technology, such as more robust quantum information storage [12,19]. Molecular spintronics, exploring spin-polarized electrical currents for information processing using molecules, could also be based on SMMs, taking advantage of the great tunability offered by supramolecular chemistry [6,10].

There are already plenty of great dedicated review articles focusing on the state-of-the-art synthesis, structural, and magnetic characterizations of SMMs [9,14]. Complementary to these topics, this review will endeavor to cover (i) the experimental synthesis and characterization in brief, (ii) the theoretical and computational perspectives of SMMs, and (iii) the promising potential of SMMs for emerging technologies including high-density information storage, opto-spintronics, quantum technology, and single-molecule toroics.

The following discussion will fall into five sections. In Section 2, state-of-the-art experimental developments will be briefly covered. In Section 2.2, we will discuss the important magnetic properties of SMMs. In Section 3, we will cover theories and simulations. In Section 4, we will present the applications of SMMs to QT. At the end, in Section 5, we will draw some general conclusions.

## 2. The Experimental Developments of SMMs

### 2.1. Chemical Synthesis and Molecular Ligand Engineering

An extensive range of SMMs have been synthesized, which indicates the great power of molecular engineering associated with Click Clack reactions in supramolecular chemistry [20].

The key principle for fabricating SMMs is that they need to have not only excellent magnetic properties but also stability under normal conditions. For example, SMMs will not decompose in the presence of atmospheric oxygen and natural humidity. This criterion is important for simplifying the production of SMM-based technologies. Supramolecular chemistry is of secondary nature, since it is most important to first determine the principles of constructing the necessary coordination units using specially created ligands. Then, using non-covalent (i.e., supramolecular) interactions will ensure the correct arrangement of SMMs in the crystalline space, effectively suppressing the intermolecular magnetic exchange interactions undesirable for this type of magnet. Also, supramolecular chemistry approaches can be useful for the monolayer application of the most promising SMM on the surface.

To fabricate SMMs containing 3d− and/or 4f− magnetic chemical elements effectively, there are two main approaches: (a) using ligands carefully designed to obtain appropriate coordination functional groups for 3d, 4f, or both metal ions, and (b) exploring co-ligands to assist the self-assembly processes to integrate lanthanide ions into existing ligands [21]. The two approaches can be called the designed assembly approach (DAA) and assisted self-assembly approach (ASA), respectively. Moreover, site-targeted reactions are another promising route to synthesize SMMs. Establishing the correlations between the molecular structure and the properties is important for the iterative predictable tuning of SMM properties by changing the ligand structure. For the 3d− electrons, the orbital overlap is dominant, while the bonding between lanthanides carrying 4f− electrons and the ligand is ionic.

In the DAA, one can design organic ligands such as Schiff-based ligands. Schiff-based ligands could provide an ideal route to fabricate SMMs as they can have distinct coordinate pockets, thus binding many kinds of metal ions. In addition, oximes and pyridonate ligands can also be used for the DAA. In addition, amino acids, alkylol amines, flexible hexa-dentate ligands, Calix[4]arenes (H4L54, bowl-shaped ligands), and hexaimine macrocyclic ligands (H6L55R, R = Pr, Ox, Ph, Bu) can be useful for the DAA. For the ASA, acetate and nitrate can bridge co-ligands, while ${\mathrm{Hfac}}^{-}$, $N_{3}^{-}$, ${\mathrm{SCN}}^{-}$, and $C_{2}O_{4}^{-}$ can work as terminal ligands. A more detailed review of the synthesis of SMMs can be found in Refs. [21,22]. Although the DAA and ASA can be used to synthesize stable structures, most coordinated structures could be detrimental to the expected SMM properties. However, highly active reagents can be used to remove unnecessary coordinated anions or solvents. The design of organic ligands compatible with the electronic structure of magnetic elements is therefore crucial.

### 2.2. Experimental Characterizations

Up to now, huge experimental efforts have been made to study the magnetic properties of SMMs, which are governed by quantum mechanics in principle. The quantum mechanical spin Hamiltonian below governs the magnetic behavior of SMMs under an external magnetic field.

(1) $$\begin{array}{ccc} \hat{H} & = & \sum_{i,j}J_{ij}{\hat{\vec{s}}}_{i}\cdot {\hat{\vec{s}}}_{j}+\sum_{i}D_{i}{\hat{s}}_{iz}^{2}+E_{i}\left({\hat{s}}_{ix}^{2}-{\hat{s}}_{iy}^{2}\right)+\sum_{i}\mu_{B}\vec{B}\cdot {\vec{\vec{g}}}_{e}\cdot {\hat{\vec{s}}}_{i}\\ & & +\sum_{i,j}A_{ij}{\hat{\vec{s}}}_{i}\cdot {\hat{\vec{I}}}_{j}+\sum_{j}\mu_{N}\vec{B}\cdot {\vec{\vec{g}}}_{N}\cdot {\hat{\vec{I}}}_{j} \end{array}$$

Here, ${\hat{\vec{s}}}_{i,j}$ (${\hat{\vec{I}}}_{i,j}$) denotes spins due to the electron (nuclear) in the SMM, $J_{ij}$ refers to the exchange interaction between spins ${\hat{\vec{s}}}_{i}$ and ${\hat{\vec{s}}}_{j}$, $A_{ij}$ is the hyperfine interaction between ${\hat{\vec{s}}}_{i}$ and ${\hat{\vec{I}}}_{j}$, $D_{i}$ and $E_{i}$ are the uniaxial and biaxial magnetic anisotropy for spin *i*, respectively, $\vec{B}$ is the magnetic field, ${\vec{\vec{g}}}_{e}$ (${\vec{\vec{g}}}_{N}$) is the electron (nuclear) *g*-tensor, and $\mu_{B}$ ($\mu_{N}$) is the Bohr magneton (nuclear magneton).

The blocking temperature ($T_{B}$) and effective tunneling energy barrier ($U_{\mathrm{eff}}$) are the crucial parameters for the magnetic properties of SMMs. Previously, $T_{B}$ was defined as the temperature at which the magnetic relaxation time is 100 s [14]. This definition provides a standard method to compare the magnetic properties of SMMs. A more flexible definition for $T_{B}$ is the temperature at which the magnetic relaxation is slower with reference to the time scale of the characterization instrument.

In addition, $T_{B}$ can be defined from alternative perspectives including thermal energy, hysteresis, magnetic susceptibility, zero-field-cooling (ZFC), field-cooling (FC) divergence, and the magnetic tunneling effect [7,23,24,25,26]. In terms of the competition between the thermal energy and the magnetic anisotropy, $T_{B}$ can be defined as the temperature below which the thermal energy can not overcome the magnetic anisotropy. This is consistent with the definition based on spin relaxation because spin relaxation is driven by thermal fluctuations. $T_{B}$ can also be defined as the highest temperature at which magnetic hysteresis can be observed. From the point of view of cooling, $T_{B}$ can be defined as the temperature where the ZFC and FC curves diverge. The peak of the out-of-phase component $\chi^{\prime \prime }$ of the temperature-dependent AC magnetic susceptibility can also refer to the blocking temperature. The temperature at which the quantum tunneling effect is negligible can also be defined as $T_{B}$.

$U_{\mathrm{eff}}$ is defined as the spin inversion energy barrier from spin-up to spin-down states, or vice versa, which is related to quantum tunneling of magnetization (QTM). Suppression of QTM is important for the further development of SMMs [27,28,29]. $U_{\mathrm{eff}}$ defines how SMMs relax at a particular temperature *T* as described in the formula $\tau^{-1}=\tau_{0}^{-1}e^{-U_{\mathrm{eff}}/k_{B}T}$. Here, τ is the thermal relaxation time, $\tau_{0}$ is the attempt time (typically between $10^{-10}$ and $10^{-9}$ s), and $k_{B}$ is the Boltzmann constant. The 3d-block SMMs show the following relationship between $U_{\mathrm{eff}}$ and the magnetic anisotropy: $U_{\mathrm{eff}}=\left|D\right|S^{2}$ and $U_{\mathrm{eff}}=\left|D\right|\left(S^{2}-\frac{1}{4}\right)$ for the integer and the half-integer spin quantum number *S*, respectively. However, the correlation between the $U_{\mathrm{eff}}$, the magnetic anisotropy, and the spin quantum number is much more complicated [9] for *f*-block SMMs. SMMs, carrying a spin angular moment, are a natural candidate for quantum information storage units. SMMs can inherently work as a qudit, where *d* refers to the dimension of the quantum information storage unit represented by the magnetic moment. For example, spin-$\frac{1}{2}$ is a qu*b*it, with two dimensions spanned by spin-up and spin-down. To assess SMMs’ properties for quantum computing, the knowledge of the spin–lattice relaxation time ($T_{1}$) and the spin–spin relaxation time $T_{2}$ is necessary. The spin–lattice relaxation time is the lifetime for the spin to survive as a classical magnet, whereas the spin–spin relaxation time is a measure of the time limit of quantum coherence.

**Figure 3:**
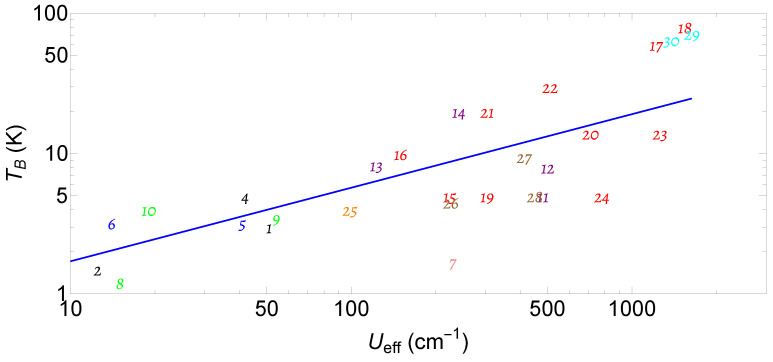
A summary (log–log plot) for the effective spin conversion barrier ($U_{\mathrm{eff}}$) and blocking temperature ($T_{B}$) for typical SMMs up to now. We have simplified the compound names to save space in the figure. Here, $1\;=\;\;{\mathrm{Mn}}_{12}$, $2\;=\;{\mathrm{Mn}}_{84}$, $3\;=\;{\mathrm{Mn}}_{6}$, $4\;=\;{\mathrm{Fe}}_{8}$, $5\;=\;{\mathrm{Mn}}^{\mathrm{II}}{\mathrm{Mo}}_{6}^{\mathrm{III}}$, $6\;=\;{\mathrm{Mn}}_{2}^{\mathrm{II}}$, $7\;=\;{\mathrm{TbPc}}_{2}^{-}$, $8\;=\;\{{\mathrm{Cu}}_{2}{\mathrm{Tb}}_{2}{\}}^{*}$, $\left.9\;=\;\{{\mathrm{Cr}}_{2}{\mathrm{Dy}}_{2}\right\}$, $10\;=\;\{{\mathrm{Cu}}_{2}{\mathrm{Tb}}_{2}{\}}^{**}$, $\left.11\;=\;[{\mathrm{Dy}}_{4}K_{2}{O(\mathrm{OtBu})}_{12}\right]$, ${12\;=\;[\mathrm{Dy}(\mu -\mathrm{OH})(\mathrm{DBP})}_{2}{(\mathrm{THF})}_{2}$, $\left.13\;=\;\{[({\mathrm{Me}}_{3}{\mathrm{Si})}_{2}N{{]}_{2}\left(\mathrm{THF}\right)\}}_{2}(\mu -\eta^{2}:\eta^{2}-N_{2}\right)$, $\left.14\;=\;[({\mathrm{Cp}}^{\mathrm{Me}4}H_{2}{\mathrm{Ln})}_{2}(\mu -N_{2}^{.}\right){]}^{-}$, ${15\;=\;\left(\mathrm{Cp}*)\mathrm{Ln}(\mathrm{COT}\right),\;16\;=\;[\mathrm{Er}(\mathrm{COT})}_{2}{]}^{-}$, $\left.{17\;=\;[\mathrm{Dy}(\mathrm{Cpttt})}^{2}\right]$, $18\;=\;[\mathrm{Dy}({\mathrm{Cp}}^{\mathrm{iPr}5}{)(\mathrm{Cp}*)]}^{+}$, $19\;=\;[\mathrm{DyZn}2({\mathrm{TTTT}}^{\mathrm{Br}}{{)}_{2}/n(\mathrm{MeOH})]}^{+}$, 20=[Dy(bbpen)Br], $21\;=\;[\mathrm{Dy}({\mathrm{OPCy}}_{3}{)}_{2}(H_{2}O{{)}_{4}]}^{3+}$, $22\;=\;[\mathrm{Dy}({\mathrm{OP}}^{t}\mathrm{Bu}({\mathrm{NHiPr}}_{2}{)}_{2}(H_{2}O{{)}_{5}]}^{3+}$, $23\;=\;[\mathrm{Dy}(O^{t}{\mathrm{Bu})}_{2}(\mathrm{py}{{)}_{5}]}^{+}$, $\left.24\;=\;[\mathrm{Dy}(L^{E}{)(4-\mathrm{MeO}-\mathrm{PhO})}_{2}\right]$, $\left.25\;=\;[{\mathrm{UO}}_{2}{\left(\mathrm{salen}\right)]}_{2}\mathrm{Mn}(\mathrm{Py}{{)}_{3}\}}_{6}\right]$, $26\;=\;[\mathrm{Fe}(C({\mathrm{SiMe}}_{3}{)}_{3}{{)}_{2}]}^{-}$, 27=[(sIPr)CoNDmp], $28\;=\;\mathrm{Co}(C({\mathrm{SiMe}}_{2}\mathrm{ONaph}{{)}_{3})}_{2}$, 29 = (${\mathrm{Cp}}^{\mathrm{iPr}5}$)_2_${\mathrm{Dy}}_{2}I_{3}$, and 30 = (${\mathrm{Cp}}^{\mathrm{iPr}5}$)_2_${\mathrm{Tb}}_{2}I_{3}$. Notice that $\left.[{\mathrm{Dy}}_{4}K_{2}{O(\mathrm{OtBu})}_{12}\right]$(11) (in purple) and $\mathrm{Co}(C({\mathrm{SiMe}}_{2}\mathrm{ONaph}{{)}_{3})}_{2}$(28) (in brown) have similar parameters, so they overlap each other. We have also added a linearly fitted line to show the trend for the optimization of the SMMs in the previous work. This trending line points to the further optimization expected in the future, going towards a higher blocking temperature and a larger effective energy barrier.

$U_{eff}$ and $T_{B}$ are important parameters for slow magnetic relaxation. More deeply speaking, the magnetic anisotropy, quantum tunneling, and the crystal field environment, especially the symmetry, play a crucial role in slow magnetic relaxation. A summary of $U_{\mathrm{eff}}$ and $T_{B}$ is illustrated in Figure 3, where we have shown polynuclear oxo-bridged transition metal SMMs (in black) [1,30,31,32], cyano-bridged SMMs (in blue) [33,34], lanthanide SIM (in pink) [35], 3d−4f SMM (in green) [36,37,38], polynuclear lanthanide SMMs (in purple) [39,40], actinide-based SMMs (in orange) [41,42], transition metal SIMs (in brown) [43,44,45], organometallic lanthanide SIMs (in red) [5,46,47,48,49,50,51,52,53,54], and a di-lanthanide SMM (in cyan) [55].

Progress has been made through continuous efforts to engineer ligands and metallic ions. The best SMM so far, the dysprosium-based lanthanide SIM, has reached a $T_{B}$ up to 80 K and a $U_{\mathrm{eff}}$ exceeding 1500 cm^−1^. Recently, there has been substantial development on combining radicals into SMM molecular structures to enhance exchange interactions and the coercive field (up to 3 Tesla) by using Ln4 metallocene complexes ($T_{B}=6.4$ K, $U_{\mathrm{eff}}=91\;{\mathrm{cm}}^{-1}$) [56]. Dysprosium (III) has also been bound with dicarbollide ligands to improve the magnetic relaxation ($T_{B}=6.8$ K, $U_{\mathrm{eff}}=559\;{\mathrm{cm}}^{-1}$) [57]. Research on SMMs also points to combining SMMs into other material structures such as metallic–organic frameworks and interesting surfaces to stabilize the SMM molecular structures, which could facilitate the design of quantum technology devices based on SMMs [58,59,60]. A high blocking temperature of 17 K ($U_{\mathrm{eff}}=427\;{\mathrm{cm}}^{-1}$) has been reported for SMMs on a graphene/Ir surface. A monolayer of SMMs on a graphene surface has impressive performance with $T_{B}=28$ K, $U_{\mathrm{eff}}=556\;{\mathrm{cm}}^{-1}$ [60]. A blocking temperature of 28 K on a surface is indeed encouraging for using SMMs as spintronic or quantum devices. More recently, di-lanthanide SMMs have been fabricated, suggesting a comparable performance to an organometallic lanthanide SIM, which points to a promising route to enhance magnetic properties by forming metal–metal bonds—an idea extended from transition metals [55]. However, the highest blocking temperature achieved so far is still far below room temperature, which needs further theoretical and experimental efforts.

**Figure 4:**
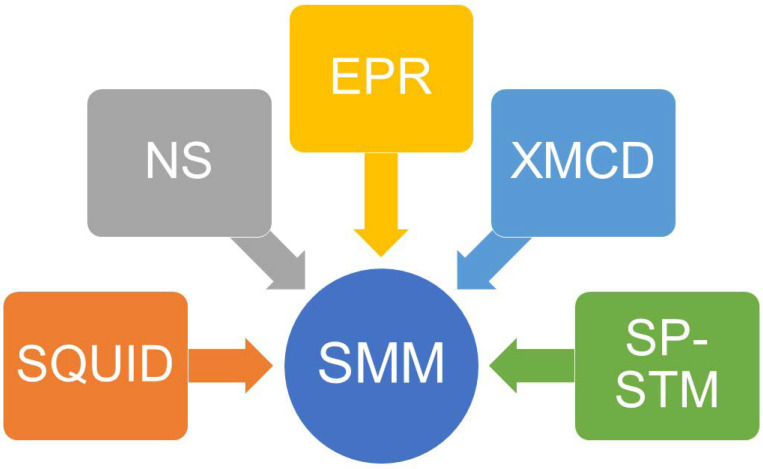
A summary of the experimental characterization methods for SMMs. SQUID = superconducting quantum interference device; NS = neutron scattering; EPR = electron paramagnetic resonance; XMCD = X-ray magnetic circular dichroism; SP-STM = spin-polarized scanning tunneling microscopy.

The experimental characterization methods have been summarized in Figure 4, and mainly include superconducting quantum interference devices (SQUIDs), electron paramagnetic resonance (EPR), neutron scattering (NS), X-ray magnetic circular dichroism (XMCD), and spin-polarized scanning tunneling microscopy (SP-STM) [12].

Observation of magnetic properties can be performed by using SQUID, which explores superconducting loops that are sensitive to a tiny change in magnetic flux. Two types of SQUID measurements are generally used, including AC and direct current (DC). AC SQUID can be used to observe the relaxation of SMMs while DC SQUID can be used for static magnetization measurements. Using DC SQUID, the magnetic exchange interaction and magnetic anisotropy can be extracted by fitting the experimental data to a spin Hamiltonian, as shown in Equation (1).

Neutron scattering (NS), using a neutron beam to probe a material’s physical properties, is a very powerful technique for studying nanoscale materials’ crystal structure, magnetic structure, magnetic excitation, spin waves, etc. [61]. Elastic NS can be used to probe static crystal and magnetic structures. Inelastic neutron scattering (INS) is another important experimental technique to capture the magnetic properties of SMMs. In INS experiments, spins exchange energy and momentum with neutrons, which allows us to probe magnetic ordering, spin waves, and magnetic excitation; these properties are closely related to exchange interactions and magnetic anisotropy. Usually, we can obtain scattering cross-sections as a function of the wave vector ($\vec{Q}$) and energy transfer (*E*), thus providing four-dimensional INS. Recently, inelastic neutron scattering experiments have been considered as a crucial tool to study SMMs [12,62].

Electron paramagnetic resonance (EPR) is a powerful tool to study the dynamics due to the interactions in SMMs, including the Zeeman energy, exchange interaction, zero-field splitting, and hyperfine interaction. The principle of EPR is to use a continuous-wave or pulsed microwave field to excite the system and record the dynamical magnetic response from it. In this way, the parameters in the spin Hamiltonian in Equation (1) can be fitted by using experimental spectra and the system is characterized. EPR is also a nice method to measure the shift and anisotropy of g-factors [63]. Nowadays, time-resolved EPR (TREPR) is applied widely to observe the spin dynamics in real time, especially under optical excitation [64,65,66]. TREPR could be a very powerful technique for spin-based quantum computing, where observing real-time spin dynamics is important for quantum operations including initialization, gate operations, and readout.

X-ray magnetic circular dichroism (XMCD) is another powerful experimental tool to observe the magnetic properties of SMMs, and it explores the difference between spectra using left- and right-circular light. XMCD is especially suitable for studying the magnetic properties of SMMs on surfaces [67,68,69,70]. The downside is that it is difficult for XMCD to address individual molecules on surfaces because it is a bulk measurement. It might be possible to develop confocal XMCD that can focus the light on a small spot such that individual molecules could be addressed optically. For this purpose, recently, nanoscale imaging for magnetic domains has been achieved experimentally [71].

Scanning tunneling microscopy (STM) was developed to use a metallic tip to probe the substances on surfaces through the tunneling electric current between the tip and surface. Spin-polarized STM (SP-STM) adds spin polarization on top of STM through a magnetically coated STM tip, which can sense magnetic substances on the surface. SP-STM would be a useful tool to study SMMs on surfaces and has the potential to effectively address individual molecules [72]. Recently, ${\mathrm{DyPc}}_{2}$ double-decker molecules have been investigated using SP-STM, and readout of spin states in different magnetic fields has been achieved [73].

Apart from the methods we discussed in detail above, there are also others used to characterize SMMs [74]. Single-crystal X-ray diffraction (XRD) and X-ray absorption spectroscopy, such as extended X-ray absorption fine structure (EXAFS) and X-ray absorption near edge structure (XANES), are effective for establishing magnetic–structural correlations [75,76]. By correlations, we mean the relationship between the influence of ligand modifications on the structure of coordination units, i.e., the parameters of magnetic anisotropy of the metal ion, and accordingly, the key physical and chemical properties of SMMs (slow relaxation of magnetization, blocking temperature, effective tunneling energy barrier, the shape of the hysteresis, and relaxation mechanisms). Reliable primary data on molecular and crystal structures from XRD and EXAFS/XANES are also very important for harnessing the full power of quantum chemical calculation methods. Electronic absorption spectroscopy (EAS) can also be used to study the electronic structure of the ground state of SMMs [77]. Moreover, luminescence spectroscopy (LS) can be employed to reveal the dependence of the energy of magnetic sub-levels on the applied magnetic field [78]. Recently, nuclear magnetic resonance (NMR) spectroscopy has also gained popularity for SMM studies [79,80]. Since NMR spectrometers are very widespread and their availability is higher than that of magnetometers, they can be an easily accessible alternative for measurements of molecular magnetic susceptibility by the Evans method and for the determination of the g-tensors, zero-field splitting energy, and ligand field parameters for SMMs in solids and solutions [79].

## 3. The Theoretical Developments of SMMs

The computation of the electronic structures of SMMs can be used to gain important insights into their magnetic properties, including exchange interactions and magnetic anisotropy, both of which are crucial to understand the magnetic properties of SMMs. To calculate magnetic anisotropy, we only need to take into account the spin–orbit interaction and spin–spin dipolar interactions for magnetic anisotropy. By contrast, we need to perform a set of calculations with distinct spin configurations to obtain the exchange interaction. Extensive computational and theoretical works have been carried out for SMMs, including studies on the electronic structure [81,82,83,84,85,86,87,88], exchange interactions [87,89,90,91,92,93,94,95,96,97,98], spin–phonon coupling [99], the effect of spin–orbit interactions [100], and relaxation rates [101,102,103,104,105,106]. A more general and comprehensive review about molecular spectroscopy is given by Ref. [107], which not only discusses the magnetic properties but also vibrational and rotational spectroscopies.

The first-principles computational methods widely used include density functional theory (DFT) [108] and quantum chemistry post-Hartree–Fock (HF) [109]. DFT is a rigorous theory that relies on charge density to predict the magnetic properties of a many-body system, whereas post-HF methods, such as configuration interaction (CI), are based on HF calculations to construct multi-determinant wave functions to obtain a better description of ground and excited states. The DFT methods include local-density approximation, generalized gradient approximation (GGA), meta-GGA, DFT + U and hybrid-exchange DFT [110,111]. As the localized *d*- and *f*-electrons are important for the magnetic properties of SMMs, we will focus our discussion on DFT+U and hybrid-exchange DFT. DFT+U, based on the conventional DFT framework, introduces a Hubbard-*U* parameter in the Hamiltonian to account for the electron correlation and localized orbitals due to d− or f− block magnetic atoms [112]. Hybrid-exchange DFT tries to mix the conventional DFT exchange–correlation functional (normally GGA) with an optimal portion of the HF exact exchange between Kohn–Sham orbitals, which is widely used to take into account electron correlations, especially in strongly correlated systems. However, the effectiveness of hybrid-exchange DFT is still controversial, which leads to the recent dedication to improve the hybrid methods or explore alternative routes [113].

The calculations of exchange interactions for SMMs, which is a challenging yet beneficial task, have been carried out predominantly by using the framework of hybrid-exchange DFT such as in Ref. [89], which is relatively efficient but on the other hand controversial in terms of calculation accuracy. Post-HF methods widely used for molecular magnets include CI, complete active space self-consistent field (CASSCF) [114], etc. CASSCF is similar to CI, but a complete active space (a set of molecular orbitals) can be defined normally near the gap between the highest-occupied molecular orbital and the lowest-unoccupied molecular orbital. An advantage of CASSCF over CI is that the molecular orbital coefficients and the CI coefficients can be determined self-consistently. The post-HF methods can provide highly accurate results but are much more expensive than DFT. In particular, the difference-dedicated configuration interaction (DDCI) can be explored to compute the exchange interaction quantitatively and analyze the mechanism for exchange interactions [115]. It is therefore very desirable to develop a theoretical framework in between, i.e., a more accurate computational method than DFT but less expensive than DDCI. Most recent work points to the decomposition method to compute exchange interactions part by part [116]. Moreover, CASSCF can also be used to compute the electronic structure, *g*-factors, and magnetic anisotropy [102,117,118,119]. CASSCF/CASPT2 (CAS-second-order perturbation theory)/RASSI-SO (restricted active space state interaction–spin orbit)/atomic mean-field approximation (AMFI) has been widely used for the computation of the parameters in the spin Hamiltonian, shown in Equation (1) [117]. The calculated results can be compared with the experiments carried out using the techniques aforementioned [117].

DFT, DDCI, and CASSCF use the same principles to compute the exchange interaction, i.e., comparing the difference in the total interaction energies with a variety of spin configurations. In fact, much of the computational time has been spent twice for the same matrix elements of either one-electron or two-electron potentials. From this point of view, Green’s function perturbation theory (GFPT) might be useful, as this theoretical frame can directly find the spin-flipping route according to the interaction *V* and propagator $G_{0}$, which is essentially similar to Feynman’s path integral formalism. However, even with GFPT methods, we still need to be careful about the accuracy of the matrix elements of the Fock matrix from either DFT or quantum chemistry calculations based on HF, which are needed for GFPT calculations.

The DFT simulations of SMMs on surfaces are particularly interesting as they are associated with a proper isolation of MMs for many useful purposes such as spintronics. Recently, simulations of this type have been carried out for coordinated lanthanide–transition-metal clusters on gold surfaces [120,121].

Another interesting research direction is to predict the optically driven exchange interaction between spins in SMMs, which can shed light on the control of molecular spins and magnetic interactions. In addition, fundamentally, this is a dynamical process involving not only the electronic state but also vibrational and rotational degrees of freedom [107].

Moreover, spin dynamics simulations within a theoretical framework of open quantum systems are becoming increasingly important [19] for a comprehensive description of how SMMs interact with the environment. The Markovian approximation for quantum dynamics, which can describe the weak coupling limit, could be useful in studying quantum systems while taking into account the perturbation from the environment [122]. This type of simulation could be useful for spin systems under external stimuli such as optical excitation. The Orbach barrier is related to the Orbach spin relaxation process involving intermediate phonon states. The Orbach barrier is then the energy needed for the Orbach process involving two phonons. Recently, a theoretical work based on the Redfield equation and non-equilibrium Green’s function method has been carried out to interpret the unexpected low Orbach barrier that is normally set by the magnetic anisotropy [86].

The software for computing the electronic structure and magnetic properties of SMMs includes Gaussian, SIESTA (Spanish Initiative for Electronic Simulations with Thousands of Atoms), ORCA, MOLCAS, CP2K, WIEN2K, Turbomole, and Amsterdam Modelling Suites (AMS) [88,123,124,125,126,127,128,129,130]. Most of these software packages use a Gaussian-type basis set, while AMS uses the Slater-type orbital [88].

## 4. Quantum Science and Technologies Based on SMMs

Quantum technologies in general include quantum sensing, quantum communication, and quantum computing. Quantum sensing, which has been performed already, is another promising direction for SMMs [131]. The quantum sensor (SMM) can interact with the external field or systems; the resulting change in spin properties can be considered the information acquired during sensing. Otherwise, the SMM quantum sensor can be embedded within the system or field under probing to obtain information about this system. Magnetic molecules can work as quantum thermometers, local temperature sensors, and local probes. In particular, molecular spin ensembles could be used to detect dark matter particles by using resonance with the effective field of axions (“cold” dark matter particles). Quantum communication relying on the excellent portability and tunability of molecular networks has also been proposed and assessed in detail [132]. Recent experimental and theoretical works have pointed to promising optically addressable spin-bearing molecules for quantum circuits and computing [10,12,19,133]. First-principles computational methods using the theory of open quantum systems have been reviewed in Ref. [133].

## 5. Conclusions and Outlook

In summary, we have reviewed the development of the research on SMMs so far and discussed their huge potential for emerging science and technology. We believe that only a tiny fraction of the literature about SMMs has been covered here, with many important works outside the scope of this paper. For chemical synthesis, we briefly cover click chemistry and supramolecular chemistry, followed by some details about the ASA and DAA methods. The experimental developments for the characterization of SMMs have been discussed briefly, including SQUID, EPR, NS, XMCD, XRD, XAFS/XANES, EAS, LS, and NMR. Here, TREPR on SMMs is particularly promising for developing quantum technology. XMCD has the potential to address SMMs on surfaces. The characterization of $T_{B}$, $U_{\mathrm{eff}}$, $T_{1}$, and $T_{2}$ parameters has also been discussed. $T_{B}$ is still much lower than room temperature, thus hindering the practical applications of SMMs. We expect more exciting research on the enhancement of $T_{B}$ in the near future. The measurements of $U_{\mathrm{eff}}$, $T_{1}$, and $T_{2}$ so far show that SMMs are very promising for emerging technologies such as quantum computing and spintronics. In addition, new theoretical techniques need to be developed to achieve more efficient computation of the exchange interaction, which is a crucial parameter for SMMs. Open quantum system simulations need to be strengthened to be able to match up with the experiments that are normally performed along with the noise from the environment. A few fascinating emerging technologies, namely high-density information storage, opto-spintronics, quantum technology, and single-molecule toroics, have been discussed. SMMs could be a good platform to realize opto-spintronics by combining the spin of the metal and the optical response of the ligand. Quantum technology is another area where SMMs could thrive as quantum information storage units for molecular networks. In summary, we anticipate an exciting era for research on SMMs.

## Data Availability

No new data were created or analyzed in this study. Data sharing is not applicable to this article.

## Nomenclature

${\mathrm{COT}}^{2}$	1,3,5,7-cyclooctatetraene
${\mathrm{Cp}}^{\mathrm{iPr}5}$	penta-iso-propylcyclopentadienyl
${\mathrm{Cp}}^{\mathrm{Me}4H}$	tetramethylcyclopentadienyl
${\mathrm{Cp}}^{\mathrm{ttt}}$	$C_{5}H_{2}^{t}$ Bu3-1,2,4
${\mathrm{Cp}}^{*}$	pentamethylcyclopentadienyl
Dmp	2,6-dimesitylphenyl
mnt	maleonitriledithiolate
$O^{t}$Bu	*tert*-butoxide
Pc	phthalocyanine
Piv	2,2-dimethylpropanoate
Py	pyridine
${\mathrm{TTTT}}^{\mathrm{Br}}$	2,$2^{\prime }$,$2^{\prime \prime }$-(((nitrilotris(ethane-2,1 -diyl))tris(azanediyl))tris(methylene))tris-(4-bromophenol)
